# Cs_2_TiI_6_ (Cs_2_TiI_x_Br_6-x_) Halide Perovskite Solar Cell and Its Point Defect Analysis

**DOI:** 10.3390/nano13142100

**Published:** 2023-07-19

**Authors:** Sadia Sultana Urmi, Md Abdul Kaium Khan, Tasnim Tareq Ferdous, Davoud Adinehloo, Vasili Perebeinos, Mohammad Abdul Alim

**Affiliations:** 1 Department of Electrical & Electronic Engineering, University of Chittagong, Chittagong 4331, Bangladesh; 2 Department of Electrical Engineering, University at Buffalo, The State University of New York, Buffalo, NY 14260, USA

**Keywords:** Cs_2_TiI_6_, DFT, point defect, lead-free, inorganic, perovskite solar cell

## Abstract

This work presents a comprehensive numerical study for designing a lead-free, all-inorganic, and high-performance solar cell based on Cs_2_TiI_6_ halide perovskite with all-inorganic carrier transport layers. A rigorous ab initio density-functional theory (DFT) calculation is performed to identify the electronic and optical properties of Cs_2_TiI_6_ and, upon extraction of the existing experimental data of the material, the cell is designed and optimized to the degree of practical feasibility. Consequently, a theoretical power conversion efficiency (PCE) of 21.17% is reported with inorganic TiO_2_ and CuI as carrier transport layers. The calculated absorption coefficient of Cs_2_TiI_6_ reveals its enormous potential as an alternative low-bandgap material for different solar cell applications. Furthermore, the role of different point defects and the corresponding defect densities on cell performance are investigated. It is found that the possible point defects in Cs_2_TiI_6_ can form both the shallow and deep defect states, with deep defect states having a prominent effect on cell performance. For both defect states, the cell performance deteriorates significantly as the defect density increases, which signifies the importance of high-quality material processing for the success of Cs_2_TiI_6_-based perovskite solar cell technology.

## 1. Introduction

Perovskite solar cell (PSC) technology is promising a breakthrough in the solar cell industry with the potential for thin-film processing, flexibility, and low-cost commercialization due to the simple solution process used in the chemical preparation of the perovskites [1,2,3]. The lead-based halide perovskites, e.g., methylammonium lead halide (MAPbX_3_) and formamidinium lead halide (FAPbX_3_), have already exceeded the recorded power conversion efficiencies of CIGS and CdTe-based solar cells [4] and, recently, a FAPbI_3_ perovskite solar cell has obtained a record power conversion efficiency (PCE) of 25.6% [5]. On the other hand, a PCE of 26.3% ± 0.5% hysteresis [6] was reported for silicon-based solar cells. As a result, perovskite solar cell technology will soon possess the potential to become a serious competitor of the well-established silicon-based solar cell technology. Presently, perovskite solar cells with higher efficiencies are mainly lead (Pb)-based due to the excellent optoelectronic properties like high carrier mobilities, long carrier lifetimes, high absorption coefficients, and direct bandgaps [7,8,9,10,11]. However, the higher efficiencies are accompanied by serious drawbacks like the toxicity of lead, reduction in the perovskite compound shelf life, and the unstable nature of device performance due to the hygroscopic and volatile organic cations [12,13,14,15]. Therefore, finding a suitable alternative for the lead (Pb)-based halide perovskites has become necessary, and extensive research is ongoing to find new perovskite materials that might yield the same high PCE or even better. Recent studies show that MA^+^ and FA^+^ can be replaced by Cs^+^ cation, leading to enhanced thermal and moisture stability [16]. Also, the toxic Pb^2+^ can be replaced by non-toxic cations like Bi^3+^, Ag^+^, Ge^2+^, Sn^2+^, In^+^, and Sb^3+^ [17,18,19]. However, the proposed lead-free perovskites suffer from very low power conversion efficiencies. In one study [17], a fabricated Cs_2_AgBiBr_6_-based perovskite solar cell could only reach an efficiency of 2.43% due to the high bandgap of the perovskite, which restricted further performance enhancement. Similar performance was observed in another [20], where an efficiency of 2.23% was achieved for the same perovskite. In a study by Chen et al. [21], the authors presented a CsSnGeI_3_-based solar cell with up to 7.11% efficiency. In another study [22], a CsSnI_3_-based solar cell achieved 6.08% efficiency, and in another [23], an efficiency of 7.5% was achieved with the incorporation of N, N′-methylenebis(acrylamide) (MBAA) with CsSnI_3_. However, the metallic conductivity of CsSnI_3_ seriously restricts the development of the solar cell [24,25], and Sn^2+^ being highly sensitive to ambient moisture and oxygen [24] makes it difficult to prevent the degradation of the solar cell.

In recent times, a new series of Ti-based lead-free vacancy-ordered halide double perovskite was proposed by Ju et al. [26] with a chemical formulation of Cs_2_TiI_x_Br_6-x_ (x = 0, 2, 4, 6). The bandgap tunability (~1.02 to ~1.78 eV) of this particular type of perovskite and the stable and nontoxic nature of Ti make them an ideal candidate for highly stable and environment-friendly single-junction as well as tandem solar cell applications. Until now, most studies have been conducted on solar cells based on Cs_2_TiBr_6_ (Cs_2_TiI_x_Br_6-x_; x = 0). Chen et al. [27] fabricated a Cs_2_TiBr_6_-based solar cell for the first time with an absorbing Cs_2_TiBr_6_ layer thickness of ~200 nm. They reported a bandgap of ~1.8 eV, consistent with the previous report [26], and the carrier-diffusion length was more than 100 nm. The solar cell exhibited high stability and the sustainability of high thermal stress in ambient conditions. However, the fabricated PSC could only achieve a stable PCE of 2.15%, which was increased to 3.22% by incorporating a C_60_ layer. To increase the PCE, a few numerical studies have been carried out [28,29,30,31,32,33,34,35,36], where different carrier transport materials were proposed for better device performance.

In the case of Cs_2_TiI_6_ (Cs_2_TiI_x_Br_6-x_; x = 6)-based solar cells, the number of studies is very limited and most of the studies lack consistency with experimental data. Previously, in one study [37], a PCE of 15.06% was reported for a Cs_2_TiI_6_ PSC with CdTe as the hole transport layer (HTL), and in another [38], a PCE of 16.31% was reported with CuSCN as HTL. However, the used bandgaps of Cs_2_TiI_6_ for these two numerical simulations are vastly inconsistent with the measured bandgap of ~1.02 eV [26], and the proposed absorber defect densities will be hard to achieve experimentally. Recently, Zhao et al. [39] provided a numerical study on Cs_2_TiI_6_ for solar cell and alpha-particle detection applications with organic PEDOT:PSS and C_60_ as carrier transport layers. They found high retainability of PCE after a very high proton fluence level and reported a PCE of 22.7% for single-junction and 26.78% for tandem solar cells. However, the high numerical PCE level was achieved for a very low defect density (10^10^ cm^−3^) of the Cs_2_TiI_6_ absorbing layer, which might be very difficult to replicate in practice because, for this family of material, the defect density level can be found in the region of 10^14^ to 10^16^ cm^−3^ and it was close to ~10^15^ cm^−3^ for the fabricated Cs_2_TiBr_6_ PSC [27,35]. Also, their simulated output of 22.7% PCE is for abnormally high electron mobility (*μ_n_* = 2.26 × 10^4^ cm^2^/Vs) and hole mobility (*μ_p_* = 7.38 × 10^3^ cm^2^/Vs), which the authors declare as an ideal case. However, these values are multiple orders of magnitude higher than what was found in practice for the same family of perovskite [27,35]. Therefore, this calls for a further investigation into the possible utilization of Cs_2_TiI_6_ as an active layer in PSCs considering the practical realizability of the device itself, and it includes making a proper choice of the carrier transport layers to maximize cell performance, even with the relatively higher defect density found in this family of perovskites. Also, Cs_2_TiI_6_-based perovskite solar cells with all-inorganic charge transport materials can be explored due to the low cost, excellent physicochemical stability, and proper photovoltaic properties of the inorganic charge transport materials [40,41]. Although the effects of different point defects on the performance of relatively high bandgap Cs_2_TiBr_6_-based PSC has been studied previously [35], there is no detailed report for the low-bandgap Cs_2_TiI_6_-based PSC. Therefore, additional insight into the existing point defects in Cs_2_TiI_6_, and the overall impact of the possible shallow and deep defect states on the performance of Cs_2_TiI_6_-based PSC, can aid experimentalists in fine-tuning material processing prior to device fabrication.

This article proposes a novel lead-free Cs_2_TiI_6_ PSC with all-inorganic carrier transport materials and an n-i-p type FTO/TiO_2_/Cs_2_TiI_6_/CuI/Au structure. At first, a first principles density-functional theory (DFT) calculation is performed to evaluate the electrical and optical properties of Cs_2_TiI_6_ and its suitability in PSC applications. Then, the device is designed using experimentally extracted data and optimized to the degree of practical feasibility. Furthermore, we analyze the impacts of existing point defects in Cs_2_TiI_6,_ and their charge transition levels on the device performance and find the keys to designing highly efficient Cs_2_TiI_6_ PSCs. We also discuss the morphological features of the material and potential ways to achieve high-quality thin films and consequently realize the full potential of the material for PSC applications. Our results demonstrate valuable insights into PSC performance, and we believe the study can accelerate/aid the practical implementation and testing of highly efficient Cs_2_TiI_6_ PSCs for different real-world applications.

## 2. Ab Initio DFT Calculation and Cell Modeling

### 2.1. First Principles Calculation of Cs_2_TiI_6_ Perovskite

**Figure 1:**
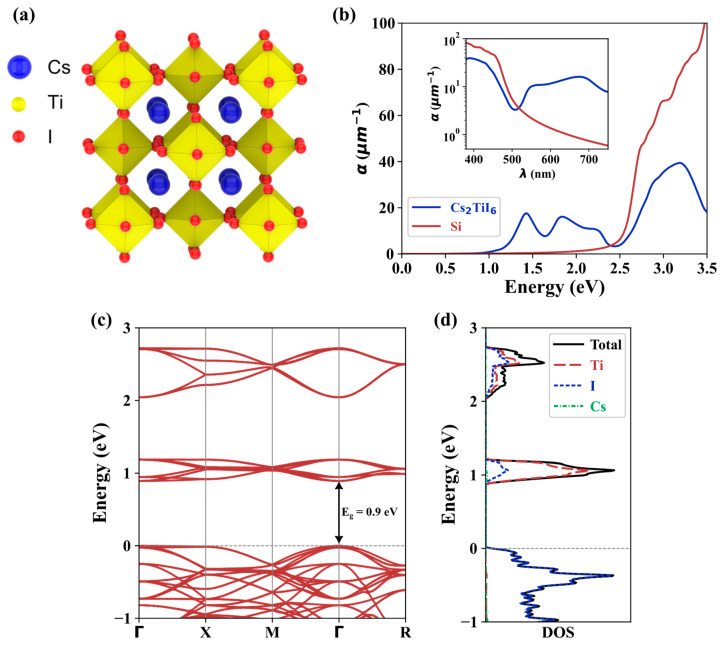
DFT calculation of Cs_2_TiI_6_: (**a**) Graphical representation of crystal structure, (**b**) computed optical absorption coefficient of Cs_2_TiI_6_ and Si, (**c**) band structure of Cs_2_TiI_6_ and (**d**) projected density of states (DOS) and total DOS of Cs_2_TiI_6_.

The electronic properties of Cs_2_TiI_6_ were calculated using the first principles DFT framework implemented in the open-source code Quantum Espresso [42]. Norm-conserving PBE pseudopotentials [43] were utilized, and the cutoff energy was set to 100 Ry. Brillouin zone sampling used an 11 × 11 × 11 k-mesh. A schematic of the crystal structure of Cs_2_TiI_6_ is shown in Figure 1a. The atomic positions were relaxed to obtain the maximum force < 0.005 eV/Å on each atom, yielding a lattice constant of 11.829 Å. The band structure of the crystal is depicted in Figure 1c. A direct bandgap of 0.9 eV was observed at the Γ point, which is slightly lower than the experimental value of ~1.02 eV [26]. However, it is worth noting that PBE is known to underestimate the bandgap, which has contributed to this discrepancy [44,45]. Also, a similar discrepancy was found for the DFT calculation of Cs_2_TiBr_6_ using PBE (E_g_~1.5 eV) and its experimental bandgap value (E_g_~1.8 eV) [27]. Figure 1d shows the density of states (DOS) and the projected DOS of Cs_2_TiI_6_. The analysis reveals that the orbitals of Ti and I atoms mainly contributed to the conduction band’s lowest energy states and the valence band’s highest energy states. Notably, the DOS of Cs atoms did not exhibit significant contributions in these energy ranges.

Optical absorption properties were essential in evaluating the suitability of materials for photovoltaic applications. The optical absorption coefficient can be determined through the calculation of the dielectric function using Equation (1) [46]:

(1) αω=ωε2nc=2ωcε12+ε22−ε112

where ω is the frequency of the incident light, c is the speed of light, n is the refractive index, and $\varepsilon_{1}$ and $\varepsilon_{2}$ are the real and imaginary parts of the dielectric function, respectively. Silicon (Si), a low bandgap material, has been the conventional material of choice for photovoltaic applications. Figure 1b shows the calculated absorption coefficient of Cs_2_TiI_6_ and Si. For lower photon energies, Cs_2_TiI_6_ possesses a promising optical absorption and should be able to absorb light with wavelengths beyond the visible spectrum. Also, in the visible spectrum [inset of Figure 1b], Cs_2_TiI_6_ showed a promising absorption for longer wavelengths. A possible implication is that Cs_2_TiI_6_ can be used as the bottom cell in tandem solar cell structures, where Si or other lead-based or organic perovskites currently find application. Thus, Cs_2_TiI_6_ has the potential to be an alternative material for highly efficient, lead-free, all-inorganic perovskite–perovskite tandem solar cell technology.

### 2.2. Cs_2_TiI_6_ Perovskite Solar Cell

We utilized the widely known Solar Cell Capacitance Simulator (SCAPS-1D 3.3.07) software developed by the Department of Electronics and Information Systems (ELIS), University of Gent, Belgium [47,48,49]. Section S1 of the Supplementary Information discusses the numerical techniques and Poisson’s equations used in SCAPS-1D. The section also discusses mathematical equations to derive other important material parameters to design solar cells in SCAPS-1D.

In Section S2 of the Supplementary Information, we validated the simulation tool’s accuracy and the reliability of the meticulously chosen device parameters by replicating the performance of an existing fabricated device of the same Cs_2_TiI_x_Br_6-x_ family. This provided the required credibility to the numerical setup as well as the numerically predicted device performance.

**Figure 2:**
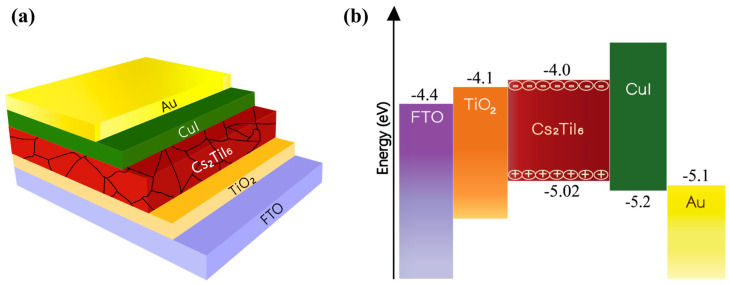
Cs_2_TiI_6_-based perovskite solar cell: (**a**) constituent layers and (**b**) corresponding energy levels.

Figure 2a is the graphical representation of different layers of the proposed device, and Figure 2b shows the corresponding energy levels. Table 1 provides the material parameters and their values used for the device modeling. The parameter values were chosen meticulously from various sources, and emphasis was given to experimentally derived data in order to design a practically realizable device. The hole transport layer (HTL) and electron transport layer (ETL) thicknesses were chosen carefully to ensure adequate light transmission through FTO onto the absorber layer. Where a thick layer might prevent enough light transmission and generation of adequate electron-hole pairs, a very thin layer might create a leakage path due to various voids and pinholes created during thin film processing, and the device would possess poor stability due to some chemisorbed hydroxides [50].

**Figure 3:**
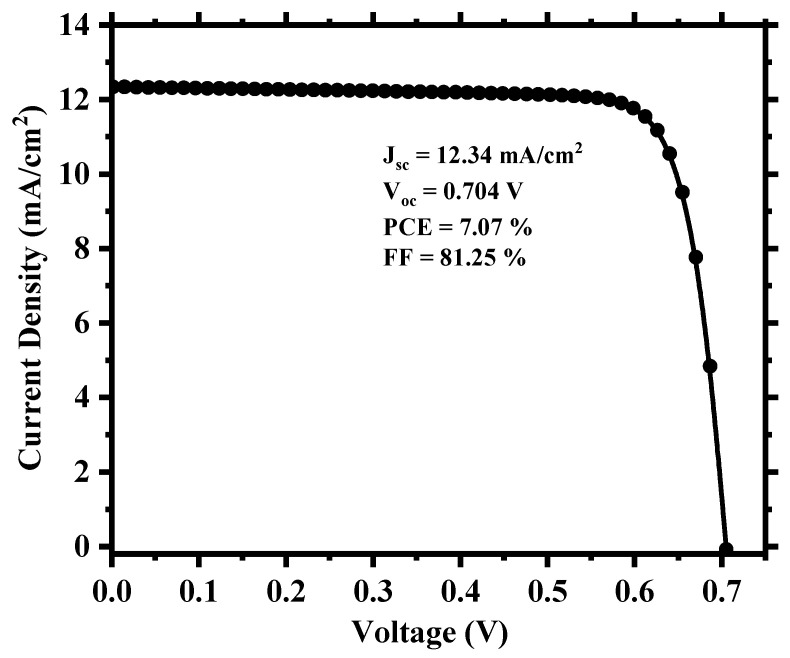
Simulated current density vs. voltage (J-V) output of the cell.

**Figure 4:**
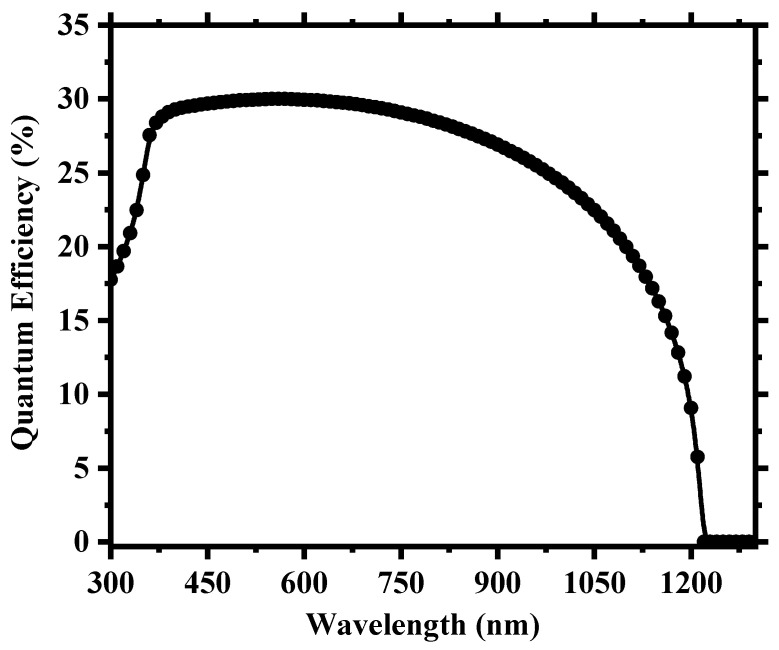
Simulated quantum efficiency (QE) as a function of wavelength for the cell.

To create a more realistic device, we applied defects in all the layers as well as in the absorber/carrier transport layer interfaces, as provided in Tables S2 and S3 of the supplementary Information. Figure 3 shows the current density (J) versus the voltage (V) output, and Figure 4 depicts the quantum efficiency (QE) versus the wavelength curve. For a high absorber defect density of 4.16 × 10^15^ cm^−3^, the device still possessed a decent PCE of 7.07% with an average quantum efficiency of ~30% for the visible spectrum. The relatively improved device performance compared to Cs_2_TiBr_6_ [27] can be attributed to the choice of hole transport material, with CuI having a very small valence band offset of ~0.18 eV and good carrier mobility that allows proper extraction and transportation of holes from the perovskite to the anode. Also, the low bandgap of Cs_2_TiI_6_ aids the creation of electron–hole pairs as it can absorb photon energy in a wide spectrum and leads to a high current density of the device. The overall impact of different inorganic hole transport layer (HTL) materials on the device’s performance is described in Section S3 of the Supplementary Information.

## 3. Cell Optimization

The proposed solar cell with existing experimentally derived parameters has already provided us with promising results. Therefore, it is necessary to extend the study further and ascertain the optimum device parameters within practically feasible limits in order to propose an optimized device to aid the experimental realization of Cs_2_TiI_6_-based perovskite solar cells.

### 3.1. Optimization of Defect Density

**Figure 5:**
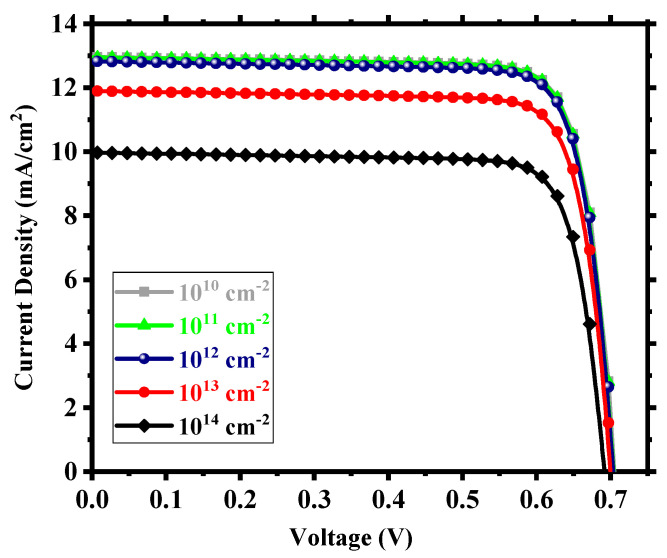
J-V output for different interfacial defect densities.

**Figure 6:**
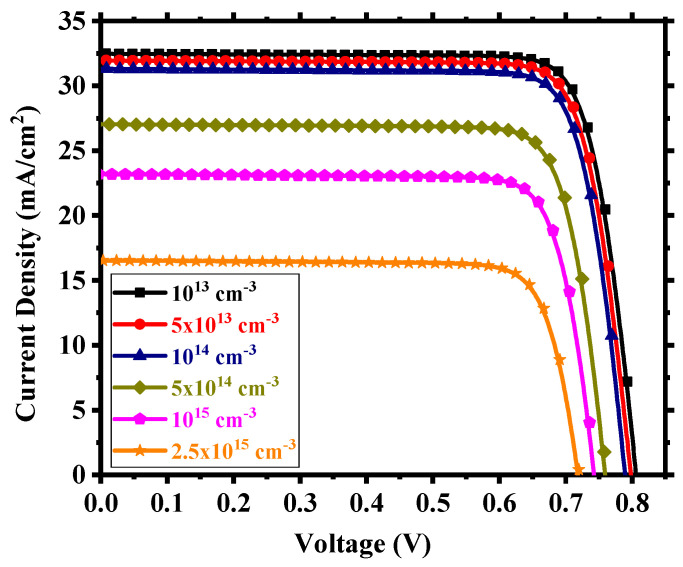
J-V output for different absorber defect densities.

We considered interfacial defects at the electron transport layer (ETL)/absorbing perovskite and absorbing perovskite/hole transport layer (HTL) interfaces as well as defects within the perovskite. The defect density within these regions is crucial for device performance. We studied the device for an interfacial defect density in the range of 10^10^ to 10^14^ cm^−2^. Figure 5 shows the corresponding J-V performance. Above 10^12^ cm^−2^, the effect of interfacial defect density became prominent with performance degradation and, below this, there was little to no change in output. The decline in performance can be attributed to the increase in the number of traps at the recombination centers [56]. For a defect density of 10^12^ cm^−2^, the device possessed power conversion efficiency (PCE) of 7.35%, fill factor (FF) of 81.25%, open-circuit voltage (V_oc_) of 0.706 V, and short-circuit current density (J_sc_) of 12.82 mA/cm^2^. With an optimum interfacial defect density of 10^12^ cm^−2^, we studied the effect of absorbing perovskite’s (Cs_2_TiI_6_) defect density in the range of 10^13^ to 2.5 × 10^15^ cm^−3^. It is quite clear from Figure 6 that the device performance relied heavily on the absorber defect density, and it was crucial to maintain an optimum defect density of this layer to achieve a high-performance PSC. Above 10^14^ cm^−3^, the device started to underperform with deterioration in performance due to the sudden expansion of recombination rate within the absorber layer [57] and, below 10^14^ cm^−3^, there was an enhancement in performance but not a substantial one. Also, we need to keep in mind the practical feasibility of achieving a certain defect profile. All aspects considered, we thus chose an absorber defect density of 10^14^ cm^−3^ as an optimum value and, with this defect profile, the device possessed J_sc_ of 31.31 mA/cm^2^, V_oc_ of 0.79 V, FF of 81.59%, and PCE of 20.22%.

### 3.2. Optimization of Cs_2_TiI_6_ Perovskite Layer

**Figure 7:**
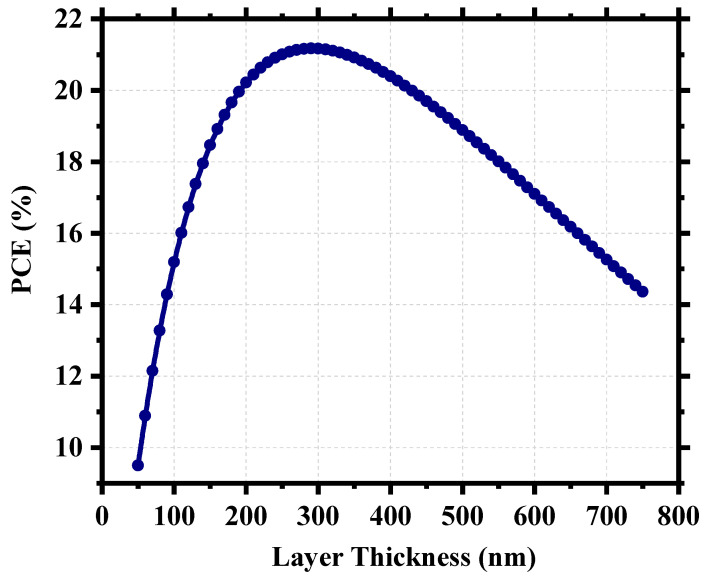
Power conversion efficiency (PCE) as a function of perovskite thickness.

For perovskite solar cells, the perovskite itself is the device’s active layer which absorbs photon energy and generates electron–hole pairs. That is why the physical properties of this layer are crucial to the overall device performance. Figure 7 shows our device’s power conversion efficiency (PCE) as a function of perovskite layer thickness and signifies the importance of having an optimized thickness. We have investigated the PCE of the device for a thickness range from 50 nm to 750 nm. For a very thin layer of perovskite, the photons with longer wavelengths face difficulties in being absorbed by the active layer [58] and, as the thickness increases, the effective bandgap withers, which aids the photon absorption [59]. In the case of a very thick layer, electrons and holes find a longer route to travel to reach the electrodes, which increases the probability of carrier recombination [52] and, as a result, the photocurrent decreases and we find a steep nature in the performance curve. The device possessed ~21% PCE when the perovskite thickness was around 300 nm, and the highest PCE was 21.18% for 290 nm. The already fabricated cell (Cs_2_TiBr_6_) of the same family had a thickness ~200 nm [27], and thus, we can choose a practically realizable thickness of 300 nm as the optimum thickness for this device with a PCE of 21.17%, J_sc_ of 32.93 mA/cm^2^, V_oc_ of 0.79 V, and FF of 81.42%.

### 3.3. Optimum Solar Cell Performance

**Figure 8:**
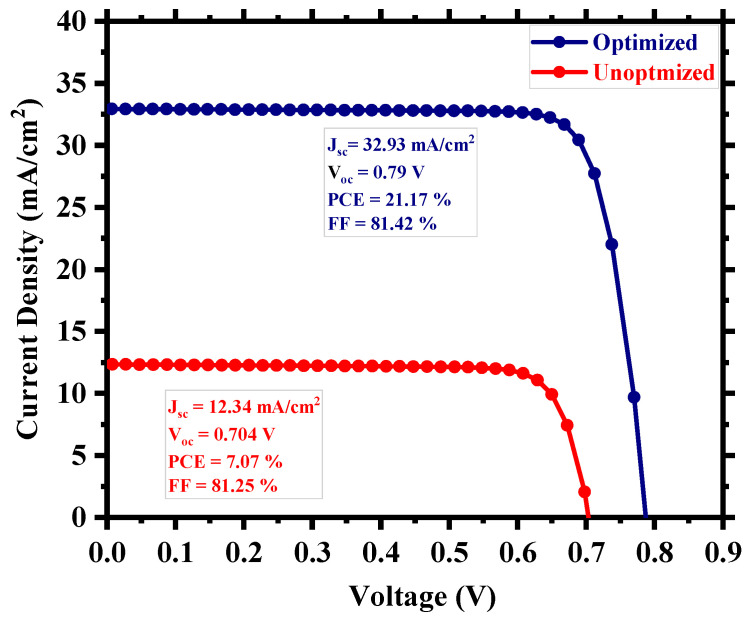
Comparison of the optimized and unoptimized cell.

**Figure 9:**
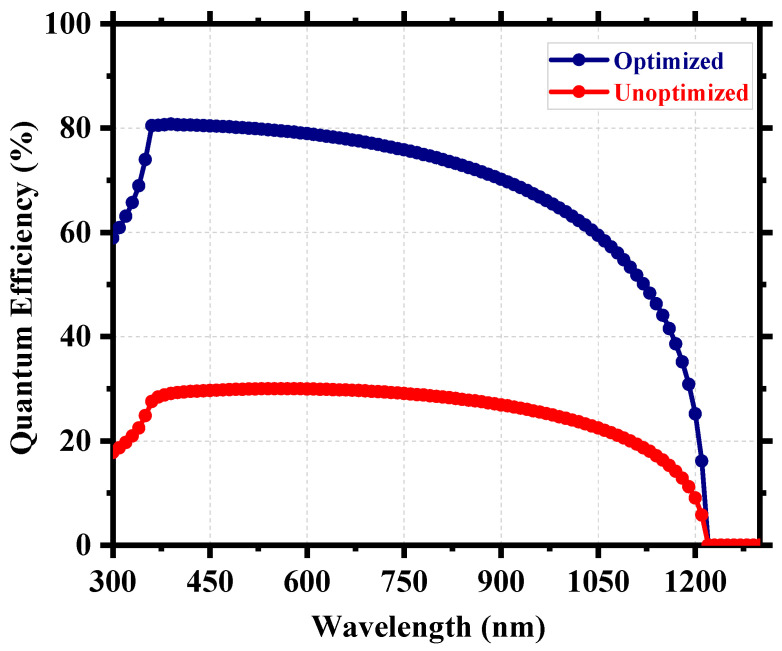
QE comparison of the optimized and unoptimized cell.

**Figure 10:**
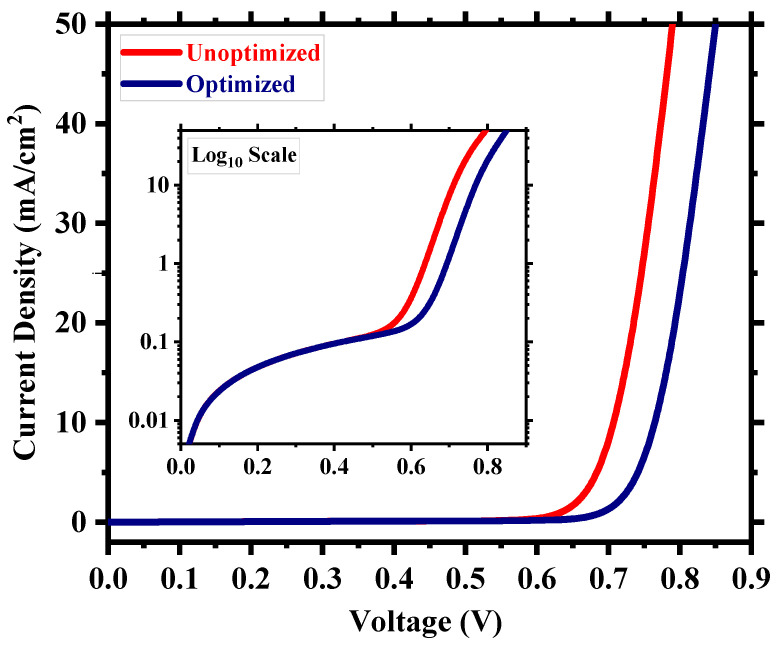
Dark current comparison of the optimized and unoptimized cell.

After the careful optimization of different device parameters, we achieved the optimum performance for a Cs_2_TiI_6_ perovskite solar cell (PSC). Figure 8 provides insight into the performance enhancement compared to the initially unoptimized state of the cell. A PCE of 21.17% with an implemented absorber defect density of 10^14^ cm^−3^ and a perovskite thickness of 300 nm was really promising. It suggests that the material processing and corresponding morphology of Cs_2_TiI_6_ thin film will be fundamentally crucial in achieving highly efficient Cs_2_TiI_6_ PSC. Figure 9 shows the quantum efficiency (QE) comparison, and Figure 10 provides the dark current comparison of the optimized final cell and the initially unoptimized cell. It can be seen that the solar cell possessed excellent QE with close to ~80% output around the visible spectrum. Also, owing to its narrow bandgap, the QE output of Cs_2_TiI_6_ existed for a wide range of wavelengths, supporting the calculation from Figure 1b, and it could have absorption cutoff up to ~1200 nm, and everything indicated that it could be a very good option as an active layer of the bottom cell in tandem solar cells. Table 2 provides a performance comparison with related numerical works on Cs_2_TiI_6_, and Table 3 shows a comparison with other PSCs from the Cs_2_TiI_x_Br_6-x_ family. As Table 2 suggests, we achieved a competitive perovskite solar cell performance despite keeping defect density and perovskite thickness within a practically realizable range.

In contrast, the previous works on this material were carried out using very low defect densities and unrealistic carrier mobilities to achieve high performance, as discussed in detail in the introduction. We achieved excellent cell performance due to our meticulous approach regarding extracting device parameters from existing experimental data, careful choice of carrier transport layers to minimize band-offset, and optimization within a feasible range. Even with a practically feasible (relatively high) defect density and standard perovskite thickness, the performance can be competitive with proper optimization of all other essential design parameters. Also, it can be easily noticed from Table 3 that most of the works on the Cs_2_TiI_x_Br_6-x_ family of perovskites are carried out using defect densities close to experimental values, and most of the absorbers’ thicknesses are around the range of ~200 nm to 400 nm with few below and above this range. Furthermore, based on the material processing, the carrier mobility might vary as well, and the possible impact of it is illustrated in Section S4 of the Supplementary Information. Figure S4 shows that the device’s PCE can vary within a range of ~19% to 24.5% for a change of one order of magnitude in carrier mobility. It is possible to enhance carrier mobility by producing high-quality thin films and, therefore, during material processing, much concentration is to be given to achieving high-quality films that might help yield even higher PCE.

## 4. Effects of Point Defects on Cell Performance

The material synthesis of Cs_2_TiI_6_ can give rise to different intrinsic point defects having defect formation energy within the material’s bandgap and acting as shallow or deep defect states. These point defects can cause performance degradation by acting as recombination centers for photogenerated charge carriers. Cs_2_TiI_6_ can have twelve possible point defects like vacancies (V_Cs_, V_Ti_, and V_I_), cation substitutions (Cs_Ti_, Ti_cs_), antisite substitutions (Cs_I_, I_Cs_, I_Ti_, and Ti_I_), and interstitials (Cs_i_, Ti_i_, and I_i_) [26]. These point defects can act as single, double, or multilevel donors or acceptors and can be located within the material’s bandgap with high or low formation energies. Ju et al. [26] calculated the formation energies of these point defects for two different cases, i.e., I-lean/Ti-rich and I-rich/Ti-lean, and identified a standard chemical potential region to have thermodynamical stability of Cs_2_TiI_6_. Table S6 in Section S5 of the Supplementary Information provides these point defects’ charge transition levels and approximate locations. In terms of formation energy, they found that under two different conditions, there were differences in the formation energies of these point defects, and there were different point defects with the lowest formation energy in each case. Under I-rich/Ti-lean conditions, V_I_ possessed the lowest energy and had a defect position (~0.57 eV) near the middle of the bandgap. This can be detrimental to the device’s performance by creating a deep defect state. Under I-lean/Ti-rich conditions, Ti_i_ possessed very low formation energy, most of its transition levels being within the mid-region of the bandgap (~0.35 eV, 0.45 eV, 0.73 eV, 0.92 eV), and can easily form a deep defect state and thus can cause some serious degradation to the device performance. There are other point defects like Cs_Ti_, which have higher formation energy in both conditions, and their defect position lies at the edge of the bandgap (~0.02 eV), and defects like these are likely to form shallow defect states and are less detrimental to the device.

**Figure 11:**
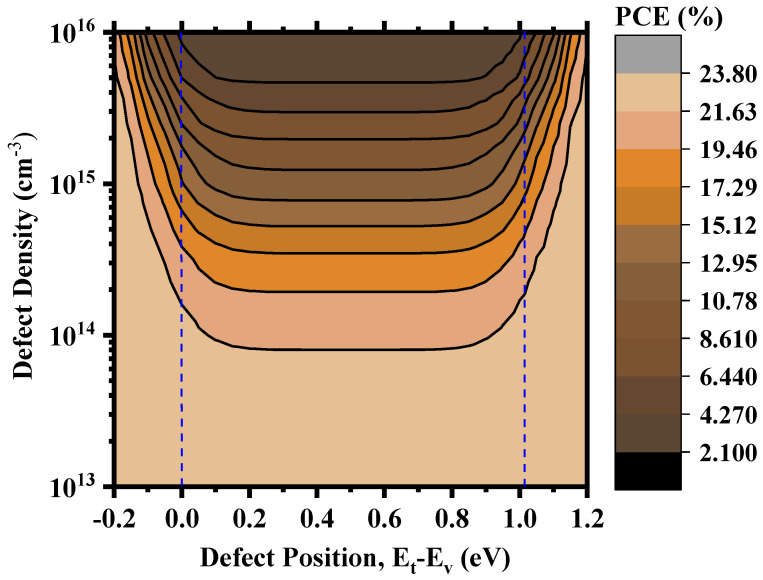
PCE as a function of defect position and defect density.

**Figure 12:**
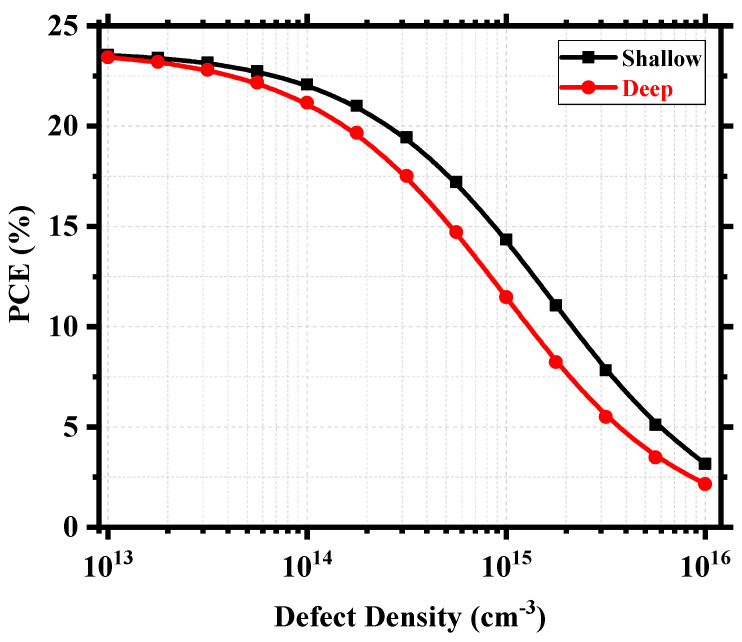
Device PCE as a function of defect density for shallow and deep defects.

Figure 11 shows an overview of the device PCE depending on the relative positions of the defects and the corresponding defect density within the perovskite. Figure S5 in the Supplementary Information shows the corresponding workflow for the calculation. We can find deep defect states within ~0.25–0.75 eV and quasi-shallow or shallow defect states around 0.0 eV (valence band maximum) and 1.02 eV (conduction band minimum). The device produced excellent PCE when the defect density was within the 10^14^ cm^−3^ mark or below, and the PCE started to decrease when the density increased towards 10^15^ cm^−3^. It still possessed a decent PCE of 11.47% around the deep defect region and 14.35% around the shallow defect region for N_t_ = 10^15^ cm^−3^. However, above 10^15^ cm^−3^, the degradation was drastic, and PCE reached as low as 2.15% around the deep defect region and 3.15% around the shallow defect region for N_t_ = 10^16^ cm^−3^. Therefore, it is rather easy to understand that the overall defect density within the perovskite is a crucial measure for the performance of Cs_2_TiI_6-_based PSCs, and anything above 10^15^ cm^−3^ will hamper the device performance significantly. The effect of deep and shallow defect states created by the point defects can be evaluated better from Figure 12. Here, we considered Cs_Ti_ with transition level ɛ (0/1−) at 0.02 eV as the shallow defect state and V_I_ with transition level ɛ (0/1+) at 0.57 eV as the deep defect state. Assuming I-lean/Ti-rich conditions are to be avoided in favor of the I-rich/Ti-lean conditions during the synthesis of Cs_2_TiI_6_ in order to suppress harmful Ti_i_ interstitial defects with very low formation energy, Figure 12 shows that, even under I-rich/Ti-lean conditions, there will be different impacts of shallow and deep defect states. With the increase in defect density, the impact becomes prominent as a higher number of V_I_ defects will act as recombination centers causing a smaller number of electron–hole pairs to reach the electrodes and, in doing so, diminishing the device performance. For example, the device PCE gets reduced by almost 3% from a PCE of 14.35% to PCE of 11.47% at 10^15^ cm^−3^ defect density when there is a presence of a deep defect state of V_I_. Therefore, we can say that the device performance heavily relies on the defect density parameter within the active layer as well as the type of point defects formed within the layer. So, the defect engineering of Cs_2_TiI_6_ will be key for the success of Cs_2_TiI_6_ PSC technology.

## 5. Discussion

The computational results suggest that defect engineering will be crucial for the success of Cs_2_TiI_6_ PSC technology. Currently, the experimental reports on the synthesis and thin film formation of Cs_2_TiI_6_ are very limited. As the defect profile is closely related to the fabrication method and the derived morphology of the thin film, it calls for concentrated experimental investigation into different morphological features and techniques to produce higher-quality thin films to suppress the negative impacts of several defects. At present, techniques like melt-crystallization [26] and inverse temperature crystallization [60] have been utilized for producing Cs_2_TiI_6_ powder and thin film (~16 μm), respectively. However, for the thin film, Cs_2_TiI_6_ solution was unevenly distributed throughout the substrate and consisted of broken cube-shaped crystals instead of a bulk perovskite layer, a representative morphology for highly efficient perovskite solar cells [61]. Another study [62] tried to generate Cs_2_TiI_6_ nanocrystals from Cs_2_TiBr_6_ nanocrystals via a post-anion exchange reaction using the hot-injection method but could not succeed. Therefore, it is evident that Cs_2_TiI_6_ has a long way to go before it can truly live up to its full potential (as expected from the theoretical study) as a high-efficiency PSC material. One important step would be to realize a stable and uniform bulk Cs_2_TiI_6_ perovskite layer, and techniques like vapor deposition [27] and fast crystallization-deposition (FDC), also known as the anti-solvent method [61], can be explored to achieve that. Extensive engineering of the experimental parameters (e.g., reaction time and temperature of vapor deposition) could lead to high-quality thin film with reduced defect profile, and further engineering of different device parameters might pave the way for Cs_2_TiI_6_ PSC technology to reach its full potential for real world applications.

## 6. Conclusions

To conclude, we have numerically demonstrated the possibility of utilizing Cs_2_TiI_6_ as an active layer in perovskite solar cell (PSC) applications with competitive cell performance. At an unoptimized state, the cell exhibits a theoretical power conversion efficiency (PCE) of 7.07% with CuI and TiO_2_ as the carrier transport layers, which is substantially increased to 21.17% for an optimum absorber defect density of 10^14^ cm^−3^ and thickness of 300 nm. Our study further highlights the importance of material processing and choosing appropriate carrier transport layers for high-performance Cs_2_TiI_6_ PSC. We have computationally demonstrated that, even with a relatively high defect density and deep defect states due to several existing point defects in Cs_2_TiI_6_, it is still possible to reach a competitive cell performance via the proper optimization of essential design parameters, subject to further enhancement depending on the improvement in defect profiles by advanced thin film processing techniques. Also, the computed electronic and optical properties of Cs_2_TiI_6_ show its great potential as an alternative low-bandgap material for different solar cell applications.

## Figures and Tables

**Table 1:** Related material parameters and their corresponding values.

Parameter	Cs_2_TiI_6_	n-TiO_2_	p-CuI	FTO
Layer thickness, *d* (nm)	200	50	50	150
Bandgap, *E_g_* (eV)	1.02	3.2	3.1	3.5
Electron affinity, *χ* (eV)	4.0	4.1	2.1	4.4
Relative permittivity, *ε_r_*	5.36	9	6.5	9
Conduction band density of states, *N_c_* (cm^−3^)	4.96 × 10^19^	1 × 10^21^	2.8 × 10^19^	2.2 × 10^18^
Valence band density of states, *N_v_* (cm^−3^)	1.75 × 10^19^	2 × 10^20^	1 × 10^19^	1.8 × 10^19^
Electron mobility, *μ_n_* (cm^2^/V s)	0.236	20	100	20
Hole mobility, *μ_p_* (cm^2^/V s)	0.171	10	43.9	10
Donor concentration, *N_D_* (cm^−3^)	3 × 10^19^	1 × 10^19^	0	1 × 10^19^
Acceptor concentration, *N_A_* (cm^−3^)	3 × 10^18^	0	3 × 10^18^	0
Thermal velocity of electron, *V_th(n)_* (cm/s)	1 × 10^7^	1 × 10^7^	1 × 10^7^	1 × 10^7^
Thermal velocity of hole, *V_th(h)_* (cm/s)	1 × 10^7^	1 × 10^7^	1 × 10^7^	1 × 10^7^
Reference	[26,27]	[27,51]	[52,53]	[54,55]

**Table 2:** Overall comparison of related numerical reports on Cs_2_TiI_6_ PSCs.

Cell Structure	Absorber Defect Density (cm^−3^)	Absorber Thickness (nm)	PCE (%)	J_sc_ (mA/cm^2^)	V_oc_ (V)	FF (%)
CuSCN/Cs_2_TiI_6_/CdS/Si [28]	-	1500	3.13	4.6	-	-
ITO/TiO_2_/Cs_2_TiI_6_/CdTe/Au [37]	-	7830	15.06	25.08	1.39	43.17
FTO/TiO_2_/Cs_2_TiI_6_/CuSCN/Ag [38]	10^10^	1000	16.31	22.74	1.74	41
FTO/PEDOT: PSS/Cs_2_TiI_6_/C60/Ag [39]	10^10^	50	22.70	39.5	0.685	83.7
**FTO/TiO_2_/Cs_2_TiI_6_/CuI/Au [This Work]**	**10^14^**	**300**	**21.17**	**32.93**	**0.79**	**81.42**

**Table 3:** Comparison with some other numerical reports on Cs_2_TiI_x_Br_6-x_ family of PSCs.

Cell Structure	Absorber Defect Density (cm^−3^)	Absorber Thickness (nm)	PCE (%)	J_sc_ (mA/cm^2^)	V_oc_ (V)	FF (%)
CuSCN/Cs_2_TiBr_6_/CdS/Si [28]	-	1000	6.68	8.9	-	-
FTO/TiO_2_/Cs_2_TiBr_6_/NiO/Au [29]	-	300	8.51	10.25	1.12	73.59
FTO/SnO_2_/Cs_2_TiBr_6_/MoO_3_/Au [30]	10^14^	130	11.49	8.66	1.53	86.45
FTO/TiO_2_/Cs_2_TiBr_6_/Cu_2_O/Au [31]	10^15^	800	14.68	25.82	1.10	51.74
ITO/NPB/Cs_2_TiBr_6_/PCBM/BCP/Ag [32]	10^17^	350	16.85	16.66	1.29	78.10
AZO/TiO_2_/Cs_2_TiBr_6_/PEDOT:PSS/Au [33]	-	200	17.83	18.20	1.38	71.00
CeO_x_/Cs_2_TiBr_6_/NPB [34]	10^15^	200	17.94	15.37	1.33	87.00
FTO/ZnO/Cs_2_TiBr_6_/MoO_3_/Au [35]	10^14^	400	18.15	13.60	1.53	87.23
FTO/BaSnO_3_/Cs_2_TiBr_6_/CuSbS_2_/Au [36]	10^13^	1000	29.13	29.60	1.11	88.58
**FTO/TiO_2_/Cs_2_TiI_6_/CuI/Au [This Work]**	**10^14^**	**300**	**21.17**	**32.93**	**0.79**	**81.42**

## Data Availability

The data that support the findings of this study are available from the corresponding author upon reasonable request.

## Supplementary Materials

The following supporting information can be downloaded at: https://www.mdpi.com/article/10.3390/nano13142100/s1. It includes five sections. Section S1: SCAPS Simulation. Section S2: Validation of Numerical Setup. Section S3: Inorganic HTL Materials. Section S4: Carrier Mobility. Section S5: Point Defects. There are six supplementary tables. Supplementary Table S1: Material parameters used to validate the numerical setup. Supplementary Table S2: Defect parameters of different layers. Supplementary Table S3: Absorber/carrier transport layer interfacial defect parameters. Supplementary Table S4: Simulation parameters of different HTL materials. Supplementary Table S5: Performance comparison of different inorganic HTL materials. Supplementary Table S6: Point defects in Cs_2_TiI_6_ with charge transitions levels and approximate defect position within the bandgap. There are four supplementary figures. Supplementary Figure S1: Simulated J-V output with experimental comparison for FTO/TiO_2_/Cs_2_TiBr_6_/P3HT. Supplementary Figure S2: J-V output for different inorganic HTL materials. Supplementary Figure S3: Relative band alignment of different HTL materials with Cs_2_TiI_6_. Supplementary Figure S4: Possible performance variation based on carrier mobility. Figure S5: Workflow for calculating PCE as a function of defect position and defect density. References [26,27,50,51,52,53,54,63,64,65,66,67] are cited in the supplementary materials.
