# Supplementary material for: Cs2TiI6 (Cs2TiIxBr6-x) Halide Perovskite Solar Cell and Its Point Defect Analysis
Source: Nanomaterials (Basel). 2023 Jul 19;13(14):2100. doi: 10.3390/nano13142100 (PMC10386147; doi:10.3390/nano13142100)
Supplement: Supplementary file 1 [file nanomaterials-13-02100-s001.zip › nanomaterials-2487464-supplementary.pdf]

Supplementary Information for

Cs_2TiI_6 ($Cs_2TiI_xBr_{6-x}$) Halide Perovskite Solar Cell and Its Point Defect Analysis

Sadia Sultana Urmi [1], Md Abdul Kaium Khan [2,], Tasnim Tareq Ferdous [1], Davoud Adinehloo [2], Vasili Perebeinos [2] and Mohammad Abdul Alim [1]*

[1] *Department of Electrical & Electronic Engineering, University of Chittagong, Chittagong 4331, Bangladesh*

[2] *Department of Electrical Engineering, University at Buffalo, The State University of New York, Buffalo, New York 14260, USA*

**Correspondence: mdabdulk@buffalo.edu*

The Supplementary Information Includes

S1. SCAPS Simulation 2

S2. Validation of Numerical Setup 3

S3. Inorganic HTL Materials 5

S4. Carrier Mobility 8

S5. Point Defects 9

References 11

S1. SCAPS Simulation

The Solar Cell Capacitance Simulator (SCAPS) solves 1D equations numerically under steady-state conditions by methods like Newton-Raphson and Gummel for convergence. The following Poisson equations are utilized for defining boundary conditions [1].

$$\frac{\partial}{\partial x}\left[\varepsilon_0 \varepsilon_r \frac{\partial}{\partial x}\phi(x)\right] = q\left[p(x) - n(x) + N_D^+ - N_A^- \rho_{def}\right] \quad \text{(S1)}$$

$$-\frac{\partial}{\partial x} J_p(x) + G(x) - R(x) = \frac{\partial p}{\partial t} \quad \text{(S2)}$$

$$-\frac{\partial}{\partial x} J_n(x) + G(x) - R(x) = \frac{\partial n}{\partial t} \quad \text{(S3)}$$

$$J_p = \frac{\mu_p}{q} p \frac{\partial E_{Fp}}{\partial x} \quad \text{(S4)}$$

$$J_n = \frac{\mu_n}{q} n \frac{\partial E_{Fn}}{\partial x} \quad \text{(S5)}$$

Here, ε_0 and ε_r are the free space permittivity and relative permittivity of the material. N_A^- and N_D^+ represents ionized acceptors and donors. $n(x)$ and $p(x)$ are the concentrations of electrons and holes. The electrostatic potential is represented by $\phi(x)$ and ρ_{def} is the charge density of the defect. $J_{n/p}$ represents the current density of electron/hole. The recombination rates and generation rates are defined by *R* and *G* respectively. $\mu_{n/p}$ represents the mobility of electron/hole, and $E_{Fn/p}$ represents the electron/hole quasi-Fermi level.

For defining material properties such as mobility (μ), total defect density (N_t), the effective conduction band density of state (N_c), and the effective valence band density of state (N_v), the following equations are utilized [2]:

$$D = \frac{l^2}{\tau} \quad \text{(S6)}$$

$$\mu = \frac{Dq}{KT} \quad \text{(S7)}$$

$$N_t = \frac{1}{\tau\sigma V_{th}} \tag{S8}$$

$$N_c = 2(\frac{2\pi m_n KT}{h^2})^{\frac{3}{2}} \tag{S9}$$

$$N_v = 2(\frac{2\pi m_h KT}{h^2})^{\frac{3}{2}} \tag{S10}$$

where D is the diffusion coefficient, l is the carrier diffusion length, τ is the carrier lifetime, K is the Boltzmann constant, T is the temperature. The capture cross-section and thermal velocity of charge carriers are defined by σ and V_{th} respectively.

S2. Validation of Numerical Setup

To increase the authenticity of the simulation setup, we have tried to validate our simulation approach by replicating the results of an experimental device from the same $Cs_2TiI_xBr_{6-x}$ family. More precisely, we tried to replicate the n-i-p type FTO/TiO_2/Cs_2TiBr_6/P3HT/Au perovskite solar cell fabricated by Chen et al. [3], where they used TiO_2 as the electron transport layer (ETL) and P3HT as the hole transport layer (HTL) and achieved a stable PCE of ~ 2.15%.

In [3], the fabricated device possesses a carrier lifetime (τ) of ~24 ns, and carrier diffusion length (l) of ~121 nm and ~103 nm for electron and hole, respectively. Other essential device parameters are derived using Equations (S6-S8). For Cs_2TiBr_6, the provided effective electron mass (m_n) of 1.79 m_e and effective hole mass (m_h) of 0.9 m_e are used in Equations (S9-S10) for calculating the conduction band and valence band effective density of states.

Table S1. Material parameters used for the validation of the numerical setup.

**Parameter**	**Cs_2TiBr_6 (Absorber)**	**FTO**	**TiO_2 (ETL)**	**P3HT (HTL)**
**Layer thickness, *d* (nm)**	200	150	50	50
**Bandgap, E_g (eV)**	1.8	3.5	3.2	2
**Electron affinity, χ (eV)**	4.0	4.4	4.1	3.2
**Relative Permittivity, ε_r**	3.75	9	9	3
**Conduction band density of states, N_c (cm^{-3})**	6×10^{19}	2.2×10^{18}	1×10^{21}	1×10^{20}

**Valence band density of states, N_v (cm^{-3})**	2.14×10^{19}	1.8×10^{19}	2×10^{20}	1×10^{20}
**Electron mobility, μ_n (cm^2/V s)**	0.236	20	20	0.0001
**Hole mobility, μ_p (cm^2/V s)**	0.171	10	10	0.0001
**Donor concentration, N_D (cm^{-3})**	3×10^{19}	1×10^{19}	1×10^{19}	0
**Acceptor concentration, N_A (cm^{-3})**	3×10^{18}	0	0	1×10^{16}
**Thermal velocity of electron, $V_{th(n)}$ (cm/s)**	1×10^{7}	1×10^{7}	1×10^{7}	1×10^{7}
**Thermal velocity of hole, $V_{th(h)}$ (cm/s)**	1×10^{7}	1×10^{7}	1×10^{7}	1×10^{7}
**Reference**	[3] [4]	[5][6]	[3][7]	[3][6]

Table S2. Defect parameters of different layers.

**Parameter**	**Absorber**	**FTO**	**ETL**	**HTL**
**Total defect density, N_t (cm^{-3})**	4.16×10^{15}	1×10^{14}	1×10^{14}	1×10^{14}
**Peak defect density (cm^{-3})**	2.35×10^{16}	5.64×10^{14}	5.64×10^{14}	5.64×10^{14}
**Energetic distribution**	Gaussian	Gaussian	Gaussian	Gaussian
**Defect type**	Neutral	Neutral	Neutral	Neutral
**Reference for defect energy level**	Above E_v	Above E_v	Above E_v	Above E_v
**Energy with respect to reference (eV)**	0.6	0.6	0.6	0.6
**Characteristic energy (eV)**	0.1	0.1	0.1	0.1
**Capture cross section of electrons (cm^2)**	1×10^{-15}	1×10^{-15}	1×10^{-15}	1×10^{-15}
**Capture cross section of holes (cm^2)**	1×10^{-15}	1×10^{-15}	1×10^{-15}	1×10^{-15}

Table S3. Absorber/carrier transport layer interfacial defect parameters.

**Parameter**	**ETL/Absorber**	**Absorber/HTL**
**Energetic distribution**	Gaussian	Gaussian
**Defect type**	Neutral	Neutral
**Total interface defect density (cm^{-2})**	5×10^{12}	5×10^{12}
**Peak interface defect density (cm^{-2})**	2.82×10^{13}	2.82×10^{13}
**Reference for defect energy level**	Above middle of interface gap	Above middle of interface gap
**Energy with respect to reference (eV)**	0.6	0.6
**Characteristic energy (eV)**	0.1	0.1
**Capture cross section of electrons (cm^2)**	1×10^{-19}	1×10^{-18}
**Capture cross section of holes (cm^2)**	1×10^{-18}	1×10^{-19}

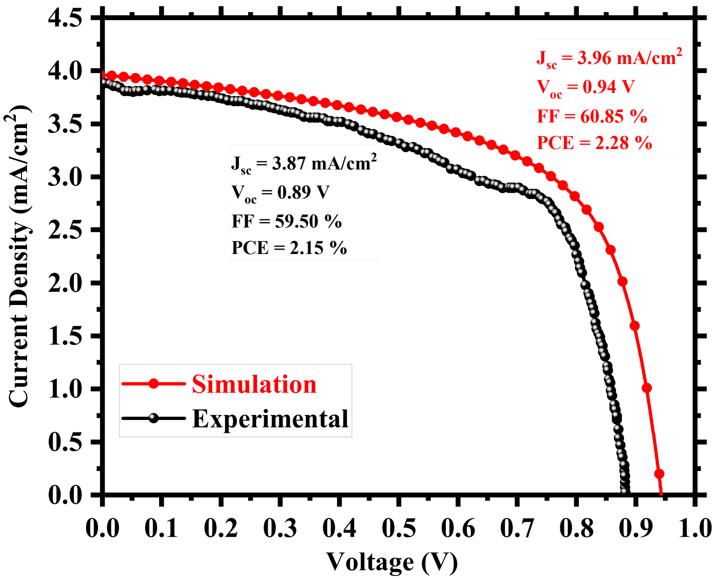

Figure S1. Simulated J-V output with experimental comparison for FTO/TiO_2/Cs_2TiBr_6/P3HT. [3].

Table S1, S2, and S3 provide all the simulation parameters for replicating the FTO/TiO_2/Cs_2TiBr_6/P3HT device. We have meticulously chosen experimental values for the device parameters and derived some of the data from experimental values using Equations (S6-S10). The standard A.M. 1.5 G spectrum is used with a constant 1000 W/m^2 illumination. The operating temperature is 300 K with 1Ω-cm^2 series resistance and 4200 Ω-cm^2 shunt resistance.

Figure S1 depicts the simulated J-V output and the replicated device's overall performance. The figure indicates that we were able to replicate the device performance to a great extent successfully. The simulated device performance is in good agreement with the fabricated device performance.

S3. Inorganic HTL Materials

We investigated different inorganic hole transport layer (HTL) materials for our device to find an ideal material with optimum performance. Table S4 provides the parameters for the investigated materials. Figure S2 shows the J-V output and Table S5 provides the performance comparison. Among all the materials, CuI possesses overall better performance. One probable reason can be the relatively better band alignment of CuI with the valence band maxima of Cs_2TiI_6. As shown in Figure S3, CuI possesses a minimum band offset of ~0.18 eV

compared to the other materials. Better band alignment accompanied with high hole mobility enhance the overall device performance and make CuI a suitable inorganic HTL material for Cs_2TiI_6-based perovskite solar cells.

Table S4. Simulation parameters of different HTL materials.

**Parameter**	**CuI**	**NiO**	**CuSCN**	**CuO_2**	**MoO_3**
d **(nm)**	50	50	50	50	50
**E_g (eV)**	3.1	3.8	3.6	2.17	3
**χ (eV)**	2.1	1.46	1.7	3.2	2.5
**ε_r**	6.5	10.7	10	7	12.5
**N_c (cm^{-3})**	2.8×10^{19}	2.8×10^{19}	2.2×10^{19}	2×10^{17}	2.2×10^{18}
**N_v (cm^{-3})**	1×10^{19}	1×10^{19}	1.8×10^{18}	1×10^{19}	1.8×10^{19}
**μ_n (cm^2/V s)**	100	12	100	200	25
**μ_p (cm^2/V s)**	43.9	2.8	25	80	100
**N_D (cm^{-3})**	0	0	0	0	0
**N_A (cm^{-3})**	3×10^{18}	3×10^{18}	3×10^{18}	3×10^{18}	3×10^{18}
**$V_{th(n)}$ (cm/s)**	1×10^{7}	1×10^{7}	1×10^{7}	1×10^{7}	1×10^{7}
**$V_{th(h)}$ (cm/s)**	1×10^{7}	1×10^{7}	1×10^{7}	1×10^{7}	1×10^{7}
**Reference**	[8][9]	[9]	[10]	[11]	[12]

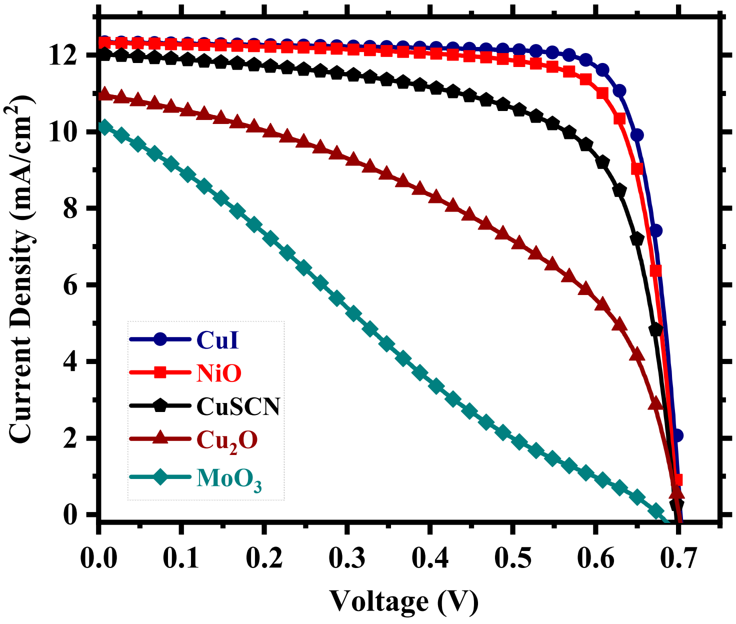

Figure S2. J-V output for different inorganic HTL materials.

Table S5. Performance comparison of different inorganic HTL materials.

HTL Material	J_{sc} (mA/cm^2)	V_{oc} (V)	FF (%)	PCE (%)
CuI	12.34	0.704	81.25	7.07
NiO	12.32	0.702	77.57	6.71
CuSCN	12.02	0.701	67.69	5.70
Cu_2O	10.98	0.703	46.93	3.62
MoO_3	10.22	0.684	23.80	1.66

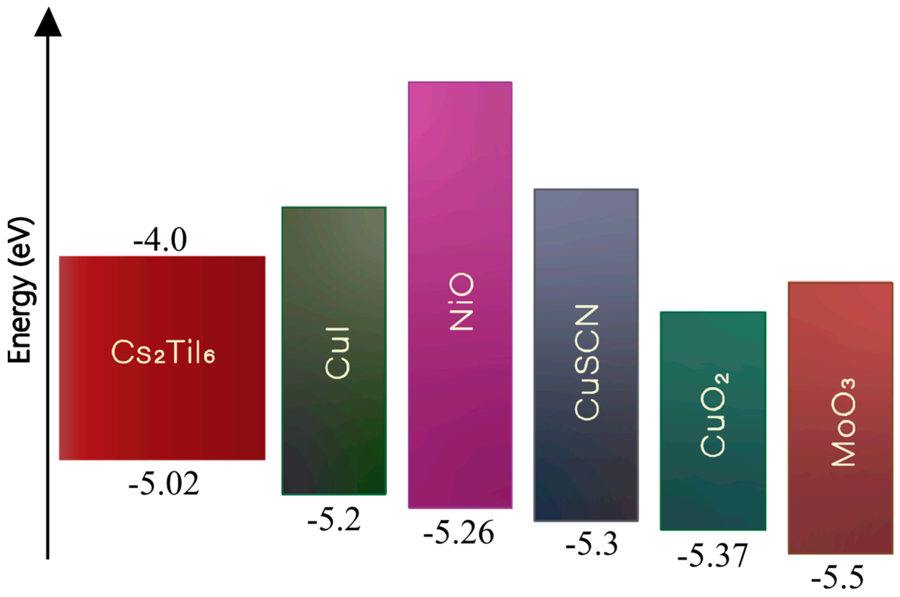

Figure S3. Relative band alignment of different HTL materials with Cs_2TiI_6

S4. Carrier Mobility

We have investigated the effects of carrier mobility on the optimized device's performance. To have a realistic scenario, we investigated the device's performance based on carrier mobility for the values within one order of magnitude of what was retrieved from experimental data of the $Cs_2TiI_xBr_{6-x}$ family. Within this range, the impact of hole mobility was prominent over electron mobility, and Figure S4 shows the effect of hole mobility variation on the device's performance. The enhancement of carrier mobility also influences the carrier diffusion length and carrier lifetime, which ultimately aids the charge collection at the electrodes and enhances the overall cell performance.

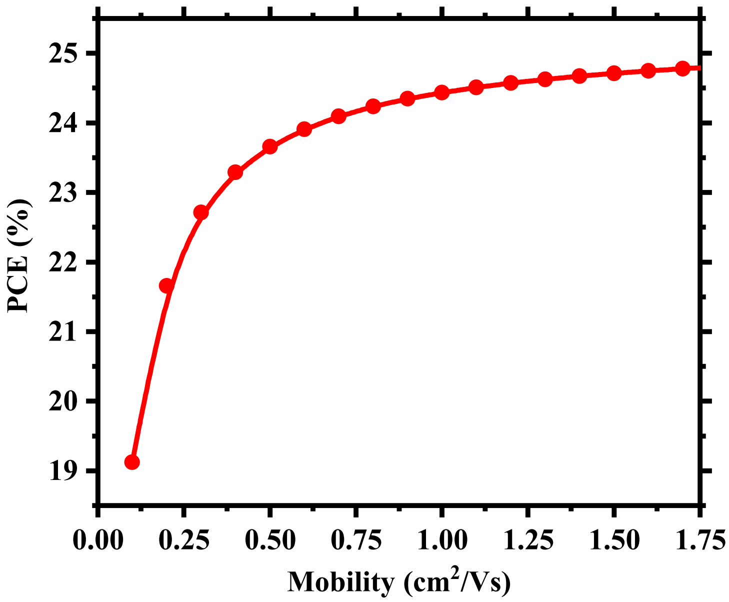

Figure S4. Possible performance variation based on carrier mobility.

S5. Point Defects

Table S6. Point defects in Cs_2TiI_6 with charge transitions levels and approximate defect position within the bandgap [4].

Types of defects	Charge transition level	Defect position above VBM (eV)
V_{Cs} (vacancy)	(0/1−)	0.83
V_{Ti} (vacancy)	(0/1−), (1−/2−)	0.5, 0.8
V_I (vacancy)	(0,1+)	0.57
Cs_i (interstitial)	(0/1+)	0.68
Ti_i (interstitial)	(0/1+), (1+/2+), (2+/3+), (3+/4+)	0.35, 0.45, 0.73, 0.92
I_i (interstitial)	(0/1−)	0.95
Cs_{Ti} (cation substitution)	(0/1−), (1−/2−), (2−/3−)	0.02, 0.18, 0.46
Ti_{cs} (cation substitution)	(0/1+), (1+/2+), (2+/3+)	0.25, 0.66, 0.73
Cs_I (antisite substitution)	(0/1+), (1+/2+)	0.55, 0.76

I_{Cs} (antisite substitution)	(0/1−), (1−/2−)	0.96
I_{Ti} (antisite substitution)	(0/1−)	0.85
Ti_{I} (antisite substitution)	(0/1+)	0.78

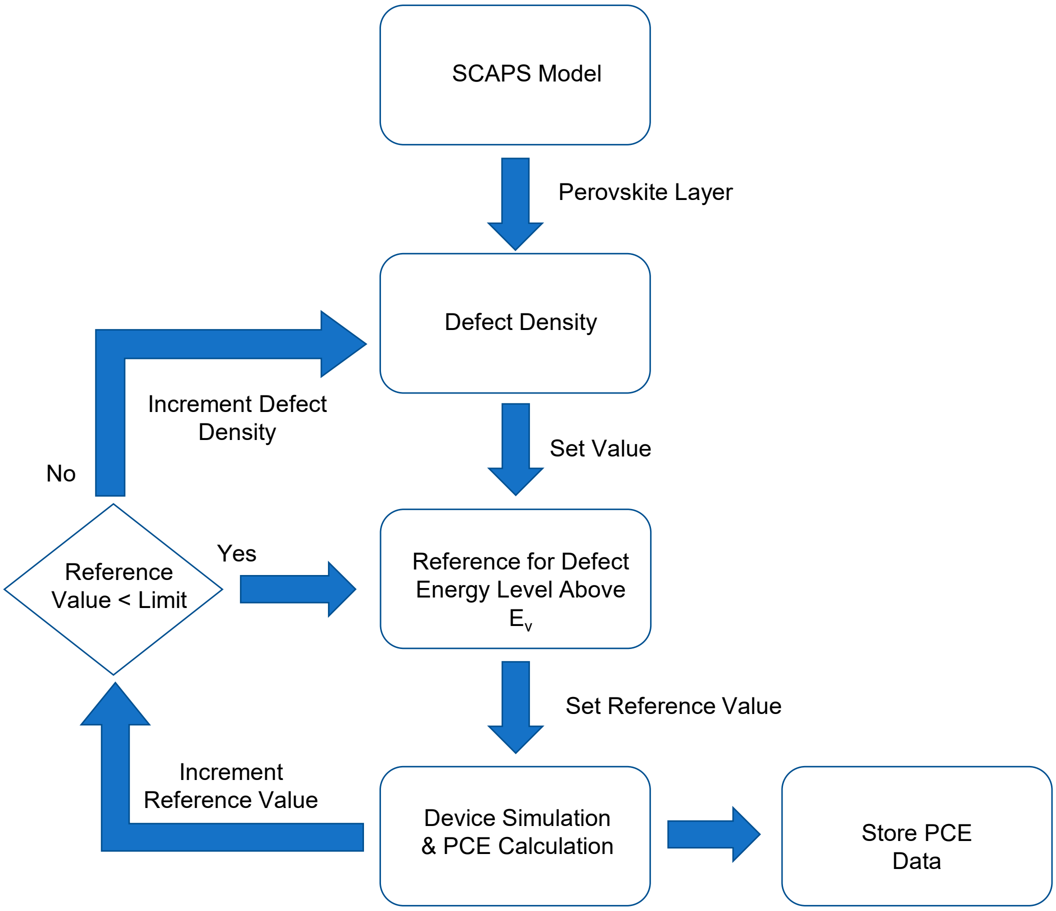

Figure S5. Workflow for calculating PCE as a function of defect position and defect density.

References

1. Bendib, T., Bencherif, H., Abdi, M. A., Meddour, F., Dehimi, L., & Chahdi, M. (2020). Combined optical-electrical modeling of perovskite solar cell with an optimized design. *Optical Materials*, *109*. https://doi.org/10.1016/j.optmat.2020.110259
2. Du, H. J., Wang, W. C., & Zhu, J. Z. (2016). Device simulation of lead-free CH3NH3SnI3 perovskite solar cells with high efficiency. *Chinese Physics B*, *25*(10). https://doi.org/10.1088/1674-1056/25/10/108802
3. Chen, M., Ju, M. G., Carl, A. D., Zong, Y., Grimm, R. L., Gu, J., … Padture, N. P. (2018). Cesium Titanium(IV) Bromide Thin Films Based Stable Lead-free Perovskite Solar Cells. *Joule*, *2*(3), 558–570. https://doi.org/10.1016/j.joule.2018.01.009
4. Ju, M. G., Chen, M., Zhou, Y., Garces, H. F., Dai, J., Ma, L., … Zeng, X. C. (2018). Earth-Abundant Nontoxic Titanium(IV)-based Vacancy-Ordered Double Perovskite Halides with Tunable 1.0 to 1.8 eV Bandgaps for Photovoltaic Applications. *ACS Energy Letters*, 3(2), 297–304. https://doi.org/10.1021/acsenergylett.7b01167
5. Helander, M. G., Greiner, M. T., Wang, Z. B., Tang, W. M., & Lu, Z. H. (2011). Work function of fluorine doped tin oxide. *Journal of Vacuum Science & Technology A: Vacuum, Surfaces, and Films*, *29*(1), 011019. https://doi.org/10.1116/1.3525641
6. Karimi, E., & Ghorashi, S. M. B. (2017). Investigation of the influence of different hole-transporting materials on the performance of perovskite solar cells. *Optik*, *130*, 650–658. https://doi.org/10.1016/j.ijleo.2016.10.122
7. Lakhdar, N., & Hima, A. (2020). Electron transport material effect on performance of perovskite solar cells based on CH3NH3GeI3. *Optical Materials*, 99. https://doi.org/10.1016/j.optmat.2019.109517
8. Yang, C., Kneiß, M., Schein, FL. et al. Room-temperature Domain-epitaxy of Copper Iodide Thin Films for Transparent CuI/ZnO Heterojunctions with High Rectification Ratios Larger than 109. *Sci Rep 6*, 21937 (2016). https://doi.org/10.1038/srep21937
9. Kanoun, A. A., Kanoun, M. B., Merad, A. E., & Goumri-Said, S. (2019). Toward development of high-performance perovskite solar cells based on CH3NH3GeI3 using computational approach. *Solar Energy*, 182, 237–244. https://doi.org/10.1016/j.solener.2019.02.041
10. F. Azri, A. Meftah, N. Sengouga, A. Meftah, (2019). Electron and hole transport layers optimization by numerical simulation of a perovskite solar cell, *Sol. Energy* 181, 372–378, https://doi.org/10.1016/j.solener.2019.02.017.
11. M.I. Hossain, F.H. Alharbi, N. Tabet, (2015). Copper oxide as inorganic hole transport material for lead halide perovskite based solar cells, *Sol. Energy* 120, 370–380, https://doi.org/10.1016/j.solener.2015.07.040.
12. W. Li, W. Li, Y. Feng, C. Yang (2019). Numerical analysis of the back interface for high efficiency wide band gap chalcopyrite solar cells, *Sol. Energy* 180, 207–215, https://doi.org/10.1016/j.solener.2019.01.018.
